# Vibration characterization of rolling bearings with compound fault features under multiple interference factors

**DOI:** 10.1371/journal.pone.0297935

**Published:** 2024-02-12

**Authors:** Yaping Wang, Huimin Yang, Songtao Zhao, Yuqi Fan, Renquan Dong

**Affiliations:** 1 Key Laboratory of Advanced Manufacturing and Intelligent Technology, Ministry of Education, Harbin University of Science and Technology, Harbin, 150080, China; 2 School of Mechanical Engineering, Tongji University, Shanghai, 201804, China; 3 Harbin Marine Boiler and Turbine Research Institute, Harbin, 150078, China; The University of British Columbia, AUSTRALIA

## Abstract

As a key component of rotating machinery power transmission system, rolling bearings in gas turbines are often required to serve in complex working conditions such as the high speed, the heavy load, the variable load, the variable rotational speed, and so on. The signals of bearing failures are easily drowned out by strong background noise and disturbances of related components. In the mechanical transmission system, the signals of bearing failures are easily submerged by the strong background noise and the disturbance of related components, especially for the composite bearing failures, which seriously hinders the effective identification of the vibration characteristics of the bearing operating state and increases the difficulty of fault diagnosis. In order to investigate the impact of interference on the bearing, through the establishment of rolling bearing composite fault vibration model, analyze the relationship between the vibration signals caused by different types of bearing failures and the corresponding vibration characteristics, to reveal the transmission system of the parts of the perturbation of the main multi-interference factors on the bearing fault signal influence law. The experimental verification shows that disturbance *y*_*p*_(*t*) caused by the sum of gear meshing frequency, and installation errors of the shaft, and coupling in the transmission system and background noise *n*_*i*_(*t*), which makes the fault frequency relatively weak and difficult to observe, and makes it difficult to accurately separate the fault information of the bearing. It provides a theoretical basis to solve the problem of damage identification and fault diagnosis of rolling bearings under multi-interference state.

## 1 Introduction

Rolling bearings, as rotations, are widely used in various fields of the modern industry. Qualities of their working conditions directly affects performances of the shafts, gears, and the entire machine equipment connected to them.

Complex and harsh working conditions often lead to failures, and early failures are often submerged in the background noise. The deterioration of the operating condition can cause related component failures, leading to bearing failure coupling and economic losses. Therefore, conducting bearing fault diagnosis under interference factors and conducting mechanism analysis research has the significant engineering application value and the practical significance [1].

The planetary gearbox on the naval gas turbine, as a transmission device, can realize the transmission of the high torque and the high speed, in which the rolling bearing is a key component in the transmission system [2]. Due to the complex structure of the transmission system, when bearing fails, the sensor required for the vibration signal monitoring can not be directly mounted on the part to be measured, but can only be mounted on the box, so the faulty bearing is not only the source of vibration itself, but also the transmission of vibration of the other parts of the system, coupled with the interference of the random noise in the industrial field, which results in the measured signal becomes unusually complex. The signal collected by the sensor after a complex transmission path not only includes the shock vibration caused by the rolling bearing fault, but also mixed with other interference components [3]. The meshing frequency of planetary gears is the main interference component, and its higher-order harmonics will submerge bearing fault information, causing great difficulties in feature extraction and diagnosis of bearing rolling element faults [4–6].

At present, the bearing fault diagnosis technology has been widely developed, but there are still problems in the research of bearing fault diagnosis, such as insufficient researches on fault symptoms and fault mechanisms, however, the fault mechanism research is an important foundation and basis for the fault prediction and safety guarantee of key equipment [7].On the basis of mechanical vibration theory, SKF of Sweden and Franklin Institute of the United States [8] cooperated to study the vibration of ball bearings, and put forward a free circular bending vibration model of bearing vibration; Li Yuqi et al [9] analyzed different initial deformation and bearing clearance by establishing a nonlinear rotor system dynamics model and solving the equations of motion of the rotor system by the Newmark-method, and then analyzed the impact of different initial deformation and bearing clearance on the nonlinear vibration of the rotor system. on the nonlinear vibration characteristics of the rotor system; Zhu Yongsheng et al [10] used the Lempel-Ziv method to quantify the bearing vibration signals and established a dynamics model containing six degrees of freedom to study the effect of compound faults on the vibration response of the bearings; Yuan et al [11] constructed a new multi-body dynamics model of the bearing system to study the evolution of compound defects and analyzed the effect of defect evolution on vibration response characteristics; Pandya et al [12] used an adaptive algorithm of wavelet decomposition and Hilbert transform to extract bearing fault feature components from vibration signals, and investigated the nonlinear dynamic response caused by compound faults; Luo Jianing et al [13,14] showed that vibration features obtained from the simulation model can provide effective sample data for the study of bearing fault diagnosis methods, and can be used as a supplement to the fault dataset (S1 Data).

In recent years, various innovative approaches have been proposed by researchers for composite fault identifications of vibration signals. Venish Suthar et al [15] applied a meta-heuristic optimization algorithm to feature vector optimization by generating additional Kurtogram images through a multiscale SinGAN model. Wang et al [16] used an integrated empirical modal decomposition (EEMD) and independent component Analysis (ICA) techniques for clearer fault identification; Sandip Kumar Singh et al [17] proposed a new method to select the Intrinsic Modal Functions (IMFs) based on the Combined Modal Functions (CMFs) and used the Convolutional Neural Network (CNN) as a deep neural network for a fault classifier. The fault information was effectively separated, which made the complex composite fault features easier to extract and clearer to recognize. Although many scholars have carried out research on vibration response characteristics of composite fault bearings, and proposed many composite fault diagnosis methods. However, the actual faulty bearings in complex working conditions will be affected by a variety of factors such as the shaft and other components and the environment, and so on. To address this problem, Yao Baihui et al [18] studied the response of the base to the bearing fault vibration signals by vibration analysis of the rotor-rolling bearing-base system, to find the base-based vibration signals of the bearing. Ning Feng et al [19] based on the proposed static mechanics, heat transfer, and friction heat generation theory of rolling ball bearings, analyzed the impact of environmental factors such as high and low temperature, alternating temperature, high vacuum, weightlessness, and strong radiation on its dynamic performance, and proposed methods and measures to eliminate or slow down the fault caused by environmental factors; Gao Tian et al [20] studied the response of the base to bearing fault vibration signals under maneuvering flight conditions, and investigated the response of the base to bearing fault vibration signals, studied the nonlinear dynamic behavior of a complex structural support-asymmetric rotor system under maneuvering flight conditions, and analyzed the vibration effects of the aircraft’s translational acceleration motion, pitch angle motion, and transient leap flight motion on the rotor and bearings.

For the detection of weak signals under the disturbed state, many scholars have proposed different methods, such as the Hilbert transform [21], the wavelet transform [22], the EMD [23], the VMD [24] and so on. Among them, Xiwen Qin [25] and others proposed a fault identification method based on the VMD and the iterative random forest classifier, which can better identify rolling bearing faults compared to other methods Tao Liang [26] and others proposed a bearing fault feature extraction method with an improved VMD parameter optimization, which can more accurately extract bearing fault features, and the fault diagnosis model based on the method has higher accuracy. The fault diagnosis model based on this method has a higher accuracy. These methods are able to suppress or eliminate the noise in the fault signal and highlight the fault features.

Nowadays, in the process of monitoring the operation status of equipment to obtain vibration signals, sensors and data transmission technologies are widely used for data acquisition, while the rapid development of simulation, data communication and other advanced technologies has triggered the rise of digital twin technology. Zhang Lei [27] used digital twin technology to construct a virtual model of the equipment, transmitted fault characteristic signals to the virtual model, and determined the reasons for the emergence of equipment faults through virtual simulation; Ye Lunkuan [28] used digital twin technology to establish a twin model of the rotating system, and compared the predicted values with the actual observed values under the data-driven approach to get the results of the equipment fault diagnosis; Hong Xuewu and et al. [29] used digital twin technology applied to ships, where the collected data is passed into the twin model to construct a fault structure tree and thus predict and evaluate the equipment state.

In summary, when establishing the bearing vibration signal response model, its vibration transmission path is first analyzed, and then the rolling bearing fault vibration response model is established from the bearing excitation point fault model. At the same time, the random interference brought by the environment, the perturbation in the transmission system dominated by the gear meshing frequency and the interaction between the faulty bearing and other components are considered. The study of above disturbing factors influences on bearing fault signals and their vibration response is a great significance for the diagnosis of bearing faults, and it provides a prerequisite guarantee for an accurate digital twin model establishment.

## 2 Rolling bearing fault characteristic frequencies

When the damage point on the working surface repeatedly contacts the surface of other components, it will cause impact vibration during the operation of rolling bearings. This periodic impact frequency is the characteristic frequency of rolling bearing component failure [30]. The fault characteristic frequencies of each part of the bearing are shown in Table 1.

**Table 1:** Formula for calculating characteristic frequency of rolling bearing.

Fault location	Characteristic frequency calculation formula
Outer ring fault	$f_{o}=\frac{Z}{2}f_{r}(1-\frac{B_{d}}{P_{d}}\cos\alpha )$
Inner ring fault	$f_{i}=\frac{Z}{2}f_{r}(1+\frac{B_{d}}{P_{d}}\cos\alpha )$
Rolling element fault	$f_{b}=\frac{P_{d}}{2B_{d}}f_{r}(1-{\left(\frac{B_{d}}{P_{d}}\right)}^{2}\cos^{2}\alpha )$
Cage fault	$f_{c}=\frac{f_{r}}{2}(1-\frac{B_{d}}{P_{d}}\cos\alpha )$

Among them, *f*_*r*_ represents the rotational frequency of the shaft (Hz), $f_{r}=\frac{R}{60}$, and *R* represents the rotational speed (r/min), *Z* represents the number of rolling elements, *B*_*d*_ represents the diameter of rolling elements (mm), *P*_*d*_ represents the pitch diameter of rolling bearings (mm), and *α* represents the pressure angle of rolling bearings. *f*_*o*_、 *f*_*i*_、 *f*_*b*_、 *f*_*c*_ indicate the characteristic frequency of rolling bearing inner and outer races, rolling elements, and cage when there is a single point of fault, respectively.

## 3 Rolling bearing compound fault vibration signal impact modeling

### 3.1 Vibration signal modeling of rolling bearing inner and outer races faults

**Fig 1:**
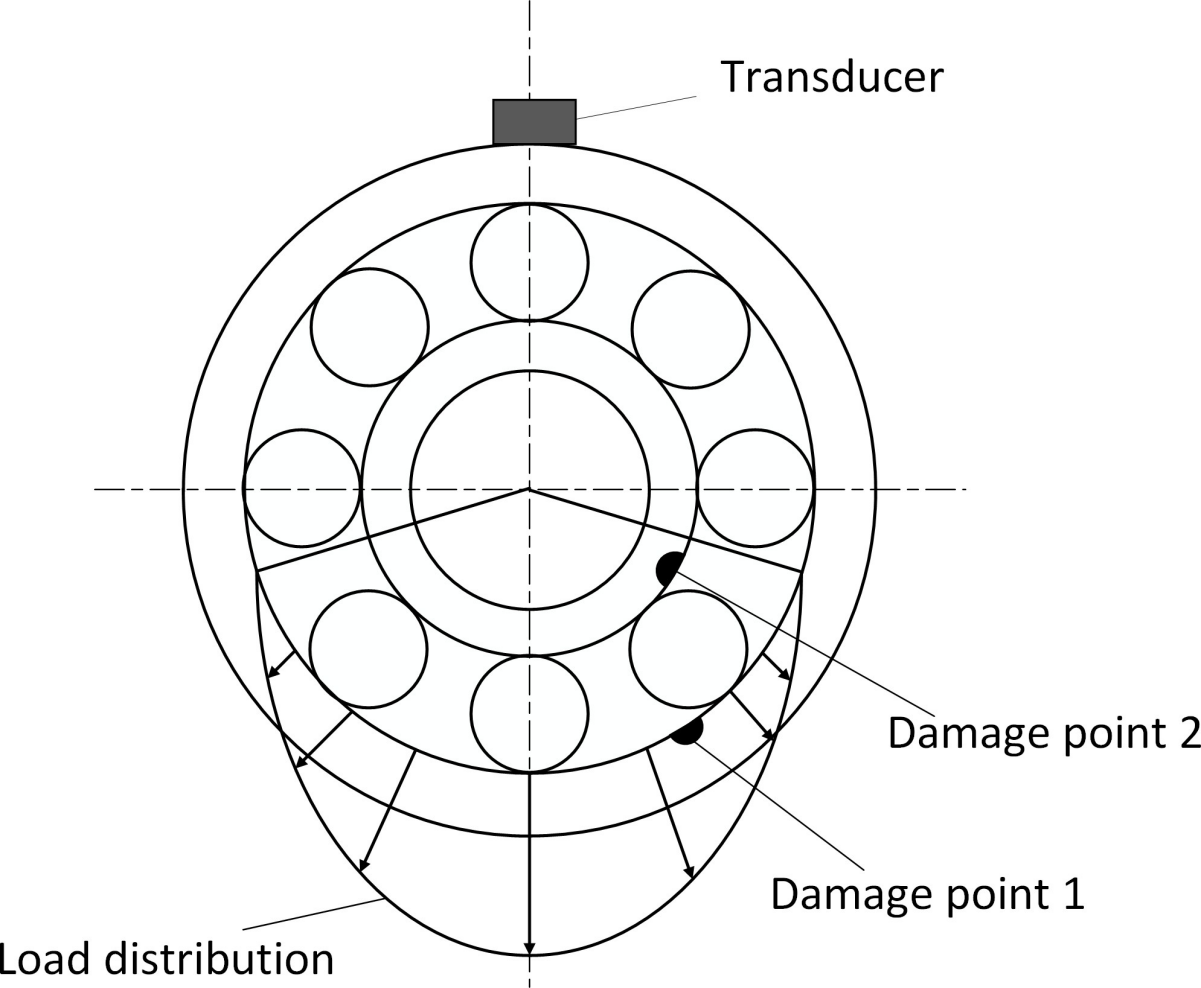
Compound fault spectrum diagram of the inner and outer races.

Compound fault spectrum diagram of the inner and outer races are shown in Fig 1. According to the literature [31], the shock vibration due to outer race fault in the frequency domain is:

$$V_{o}(f)=\Delta_{o}(f)E(f)$$ (1)

The shock vibration due to inner race fault in the frequency domain is:

$$V_{i}(f)=[\Delta_{i}(f)*C_{i}(f)]E(f)$$ (2)

**Fig 2:**
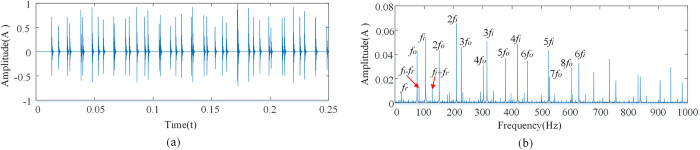
Compound fault spectrum diagram of the inner race and outer race. **(**A)Time domain diagram of inner and outer ring compound fault. (B) Composite fault spectrum diagram of inner and outer ring.

Since shocks due to the outer race are non-zero only at *f* = *kf*_*o*_, shocks due to the inner race are non-zero only at *f* = *mf*_*i*_+*nf*_*r*_. By the definition of *f*_*i*_ and *f*_*o*_, *f*_*i*_+*f*_*o*_ = *zf*_*r*_,the fault signal is compound at the above conditions. Considering that *m*, *n* and *k* are positive integers, the inner, and outer race compound fault signals are characterized by both inner and outer races fault signals as shown Fig 2.

### 3.2 Vibration signal modeling of rolling bearing inner race and rolling element faults

**Fig 3:**
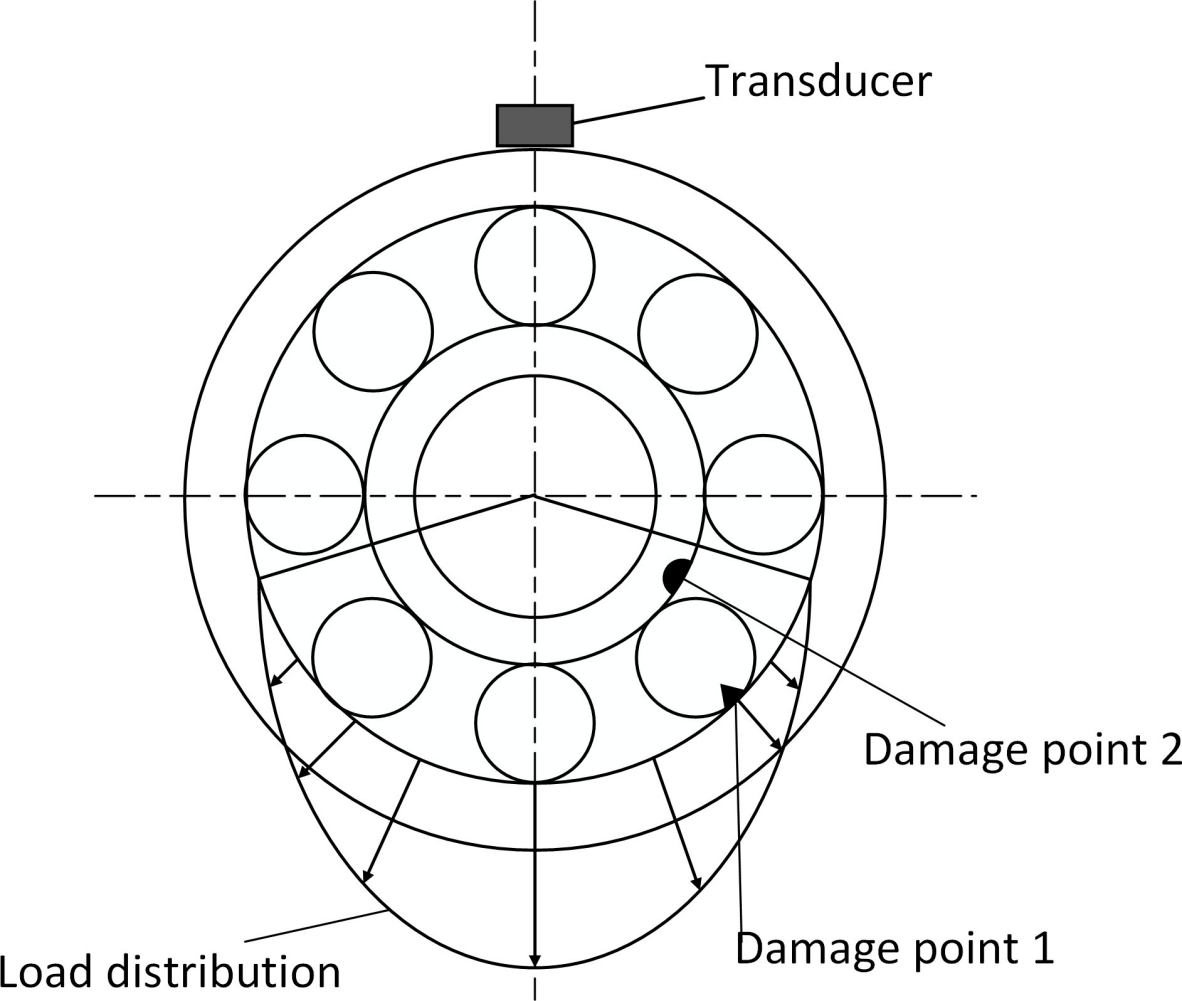
Compound fault spectrum diagram of the inner race and rolling element.

Compound fault spectrum diagram of the inner and outer races are shown in Fig 3. According to the previous section, the shock vibration caused by the inner race fault in the frequency domain is:

$$V_{i}(f)=[\Delta_{i}(f)*C_{i}(f)]E(f)$$ (3)

The shock vibration due to rolling body fault in the frequency domain is

$$V_{b2}(f)=A_{b}[\Delta_{bo}(f)*C_{b}(f)]E(f)+A_{b}[\Delta_{bi}(f)*C_{b}(f)]E(f)$$ (4)

**Fig 4:**
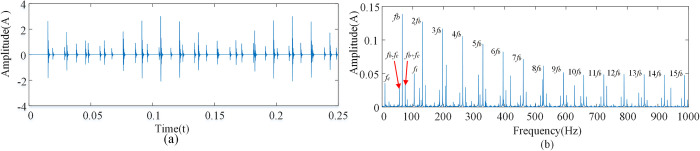
Compound fault spectrum diagram of the inner race and rolling element. **(**A)Time domain diagram of compound fault of inner ring and rolling element. (B) Composite fault spectrum diagram of inner ring and rolling element.

Shocks due to the inner race are non-zero only *f* = *mf*_*i*_+*nf*_*r*_. Shocks due to rolling elements are non-zero only = *mf*_*b*_+*nf*_*c*_. Therefore, the fault signals are compound at the above conditions. Considering that *m*, *n*, and *k* are positive integers, the compound fault signals of the inner race and the rolling element have the characteristics of both the fault signal of the inner race and the signal of the rolling element as shown Fig 4.

### 3.3 Rolling bearing outer race and rolling element fault vibration signal modeling

**Fig 5:**
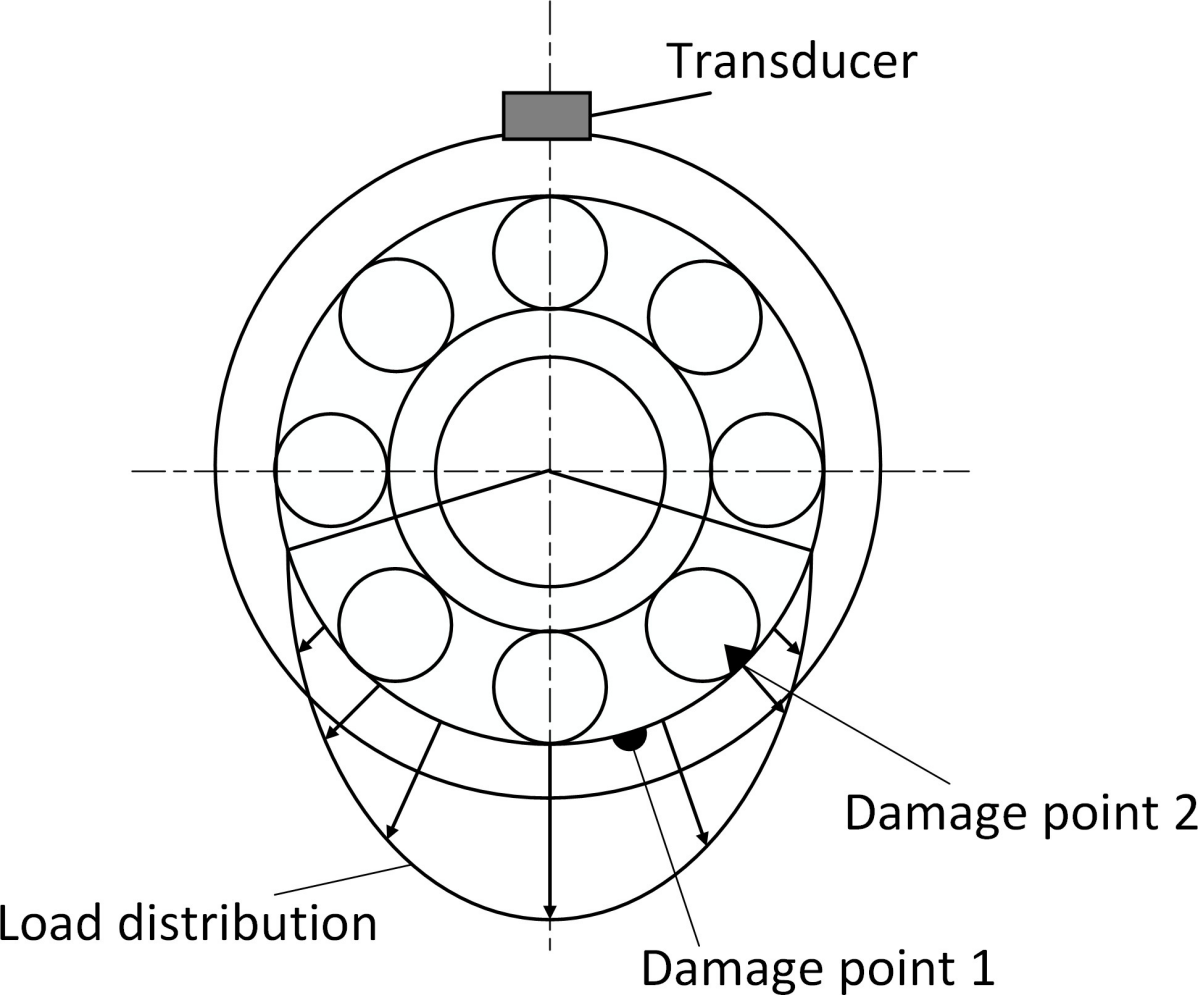
Compound fault spectrum diagram of the outer race and rolling element.

Compound fault spectrum diagram of the inner and outer races are shown in Fig 5. According to the previous analysis, the shock vibration caused by the outer race fault in the frequency domain is:

$$V_{o}(f)=\Delta_{o}(f)E(f)$$ (5)

The shock vibration due to rolling body fault in the frequency domain is

$$V_{b}(f)=A_{b}[\Delta_{bo}(f)*C_{b}(f)]E(f)+A_{b}[\Delta_{bi}(f)*C_{b}(f)]E(f)$$ (6)

**Fig 6:**
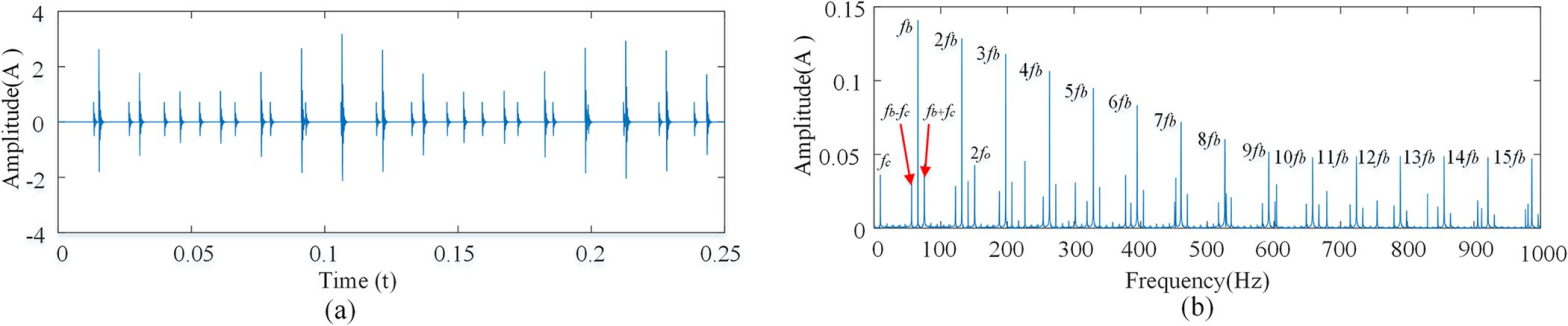
Compound fault spectrum diagram of inner race and rolling element. **(**A)Time domain diagram of compound fault of outer ring and rolling element. (B) Composite fault spectrum diagram of outer ring and rolling element.

Since shocks caused by the outer race are only non-zero at *f* = *kf*_*o*_, shocks caused by the rolling element are only non-zero at *f* = *mf*_*b*_+*nf*_*c*_. Therefore, the fault signals are compound at the above conditions. Considering that *m*, *n*, and *k* are positive integers, the compound fault signals of the outer race and rolling element have the characteristics of both the outer race fault signal and the rolling element fault signal as shown Fig 6.

### 3.4 Rolling bearing inner and outer races and rolling element fault vibration signal modeling

**Fig 7:**
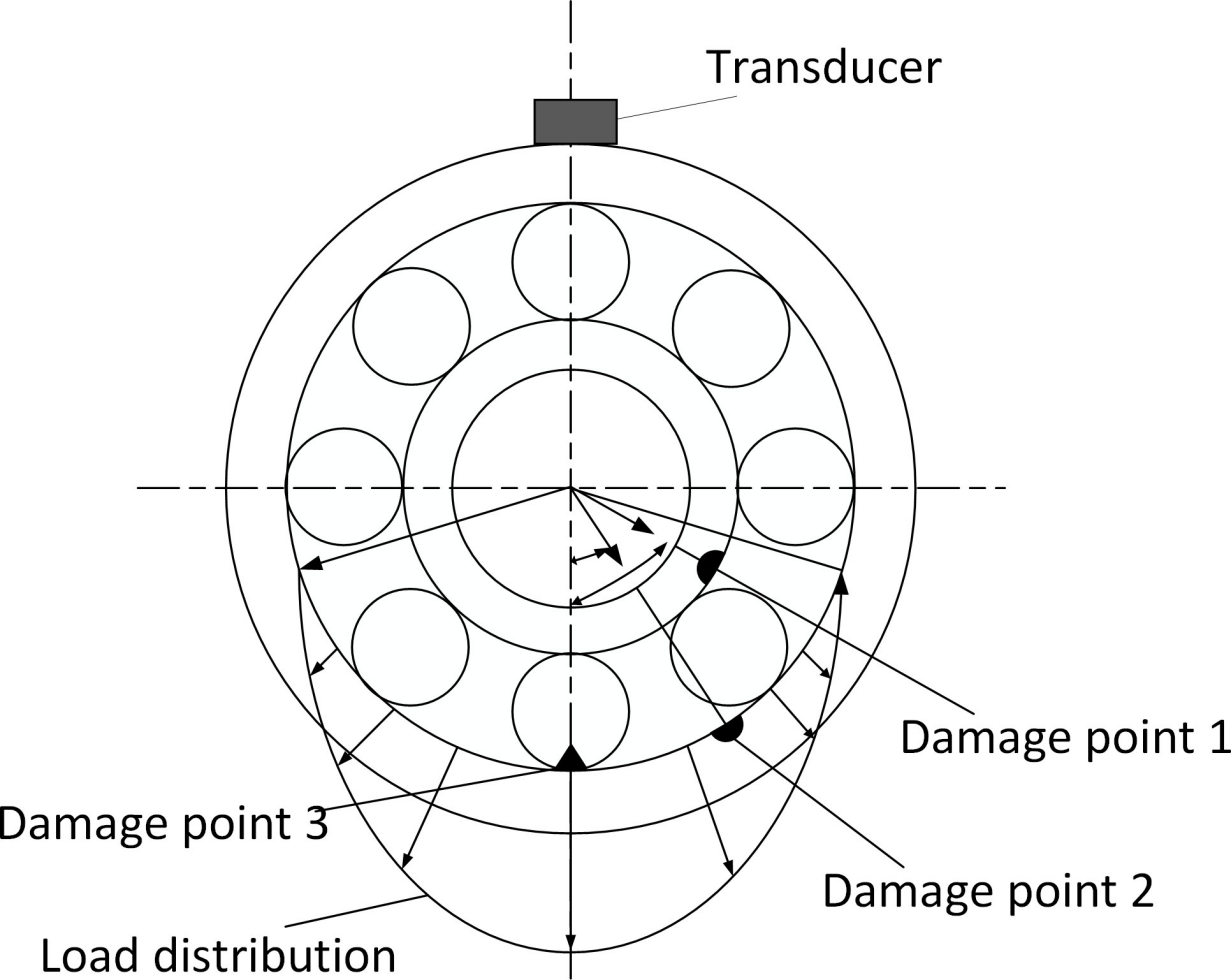
Compound fault spectrum diagram of the inner and outer races and rolling element.

Compound fault spectrum diagram of the inner and outer races are shown in Fig 7. According to the analysis in the previous section, the shock vibration caused by the outer race fault in the frequency domain is

$$V_{o}(f)=\Delta_{o}(f)E(f)$$ (7)

The shock vibration due to inner race fault in the frequency domain is

$$V_{i}(f)=[\Delta_{i}(f)*C_{i}(f)]E(f)$$ (8)

The shock vibration due to rolling body fault in the frequency domain is

$$V_{b}(f)=A_{b}[\Delta_{bo}(f)*C_{b}(f)]E(f)+A_{b}[\Delta_{bi}(f)*C_{b}(f)]E(f)$$ (9)

**Fig 8:**
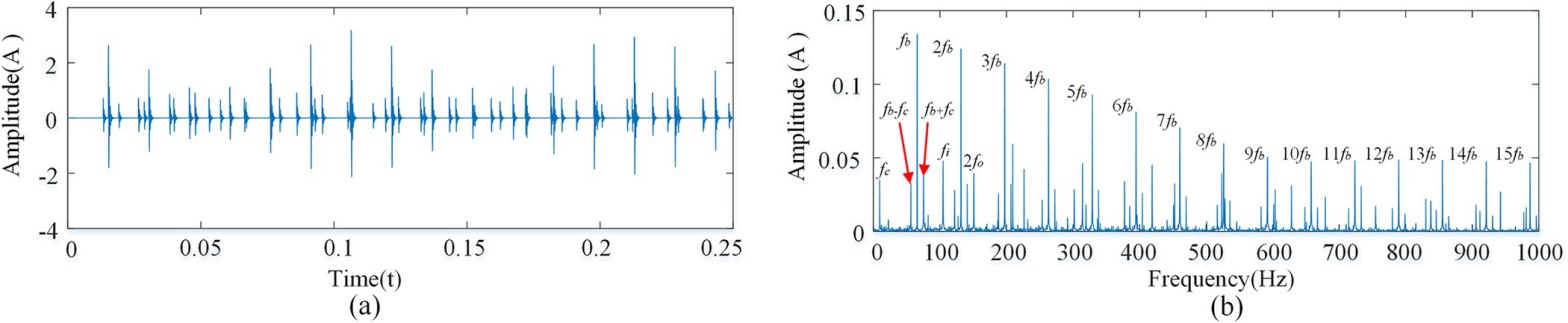
Single point fault diagram of the inner and outer races and rolling element. (A) Time domain diagram of compound failure of inner, outer ring and rolling element. (B) Composite fault spectrum diagram of inner, outer ring and rolling element.

Since the shock caused by the outer race is non-zero only at *f* = *kf_o_*, the shock caused by the inner race is non-zero only at *f* = *mf*_*i*_+*nf*_*r*_, and the shock caused by the rolling element is non-zero only at *f* = *mf*_*b*_+*nf*_*c*_. By the definitions of *f*_*i*_, and *f*_*o*_, the fault signal is compound at all points that satisfy the above conditions. Considering that *m*, *n* and *k* are positive integers, the compound fault signals of the inner and outer races and the rolling body have the characteristics of the fault signals of the inner and outer races and the fault signals of the rolling body at the same time as shown Fig 8.

## 4 Experimental signal analysis

### 4.1 Experimental environment

Structural characteristics of gas turbines combined with the comprehensive fault simulation experimental platform of rolling bearings and planetary gearboxes can effectively simulate the vibration and impact signal characteristics of rolling bearing faults in transmission systems under multiple interference factors.

**Fig 9:**
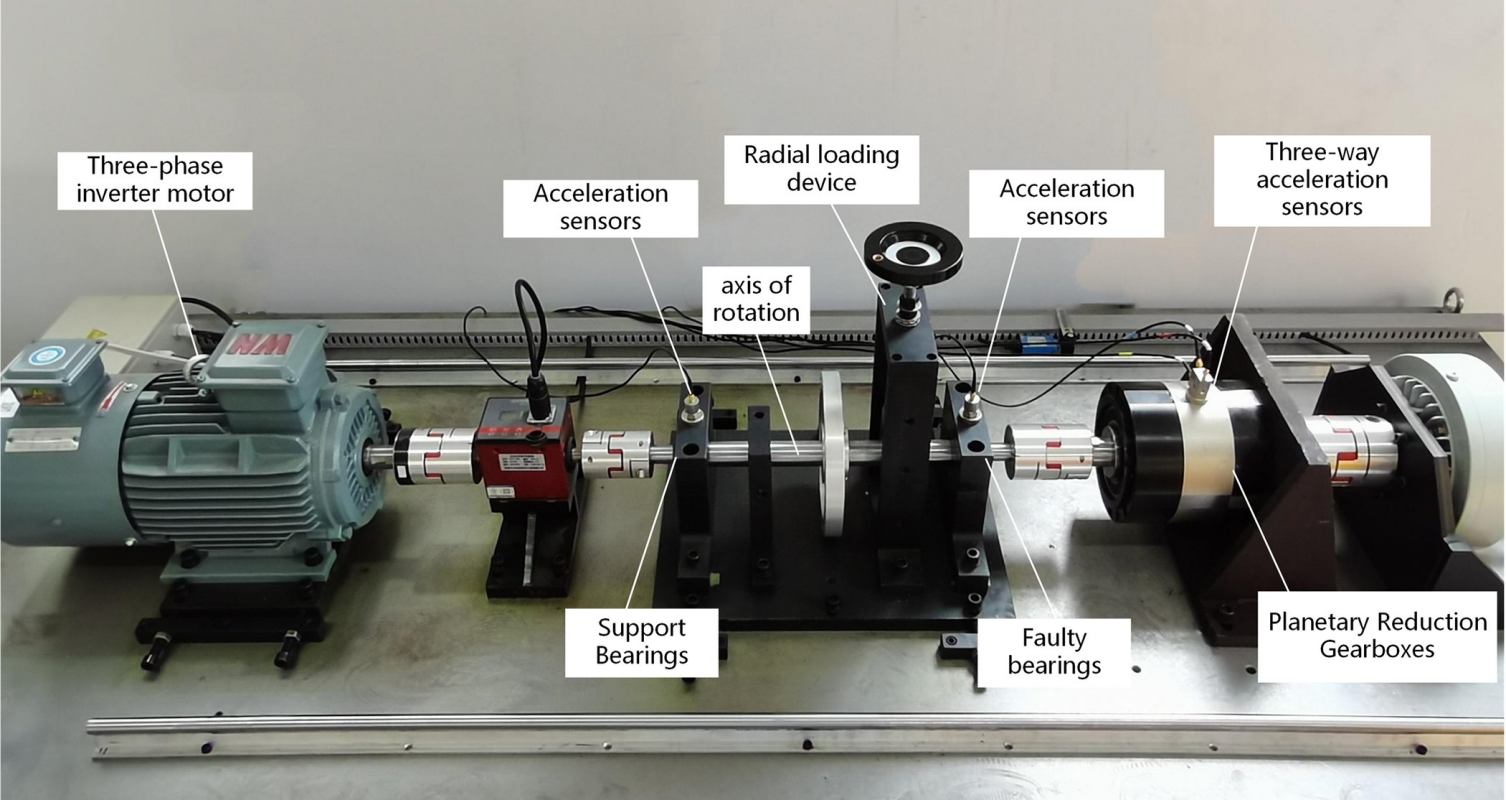
Integrated fault simulation test bench of rolling bearing and planetary gearbox.

The experimental system consists of a three-phase variable frequency motor, supporting bearings and bearing seats, tested bearings and bearing seats, radial loading device, sensor mounting bracket, planetary reduction gearbox, etc., as shown in Fig 1. the experimental system by the prime mover three-phase frequency conversion motor, support bearings and bearing housings, the measured bearings and bearing housings, radial loading device, sensor mounting bracket, the planetary reduction gearbox and so on, as shown in Fig 9.

The bearing under test is a 6205 SKF deep groove ball bearing near the gearbox. Faulty bearings made by laser cauterization of the bearing outer race, bearing inner race, and rolling element pitting compound. Vibration measurement points were the support bearing, the fault bearing where the bearing housing surface and the gearbox case shell surface, using a piezoelectric acceleration sensor. The vibration signals of the bearings and the vibration signals of the corresponding gearboxes are collected respectively.

Each type of fault is collected in 20 groups, each group contains 12,000 sample points, and the sampling frequency is set to 12 kHz. The specific parameters of the bearings and the characteristic frequency of the fault are listed in Tables 2–4.

**Table 2:** Bearing specific parameters and failure characteristics frequency table.

Parameters	Value
External diameter/*mm*	52
Internal diameter/*mm*	25
Section diameter/*mm*	38.5
Number of scrollers	9
Rolling body diameter /*mm*	7.938
Contact angle /˚	0
Frequency conversion *f*_*r*_/*Hz*	33.3
Inner race fault frequency *f*_*i*_/*Hz*	180.3
Outer race fault frequency *f*_*o*_/*Hz*	119.7
Rolling element fault frequency *f*_*b*_/*Hz*	78.7
Cage frequency *f*_*c*_/*Hz*	13.667
Number of revolutions per minute */r/min*	2000

**Table 3:** Planetary gearbox structure parameters.

Gear Category	Number of Teeth
Solar plexus chakra	21
Planetary wheel (Quantities)	31 (3)
Gear ring	84

**Table 4:** Planetary gearbox failure characterization frequency.

Parameters	Value
Solar rotation frequency *f*_*s*_/*Hz*	33.3
Planetary rotation frequency *f*_*p*_/*Hz*	20
Planetary rack frequency conversion *f*_*t*_/*Hz*	6.667
Meshing frequency *f*_*mesh*_/*Hz*	560

**Fig 10:**
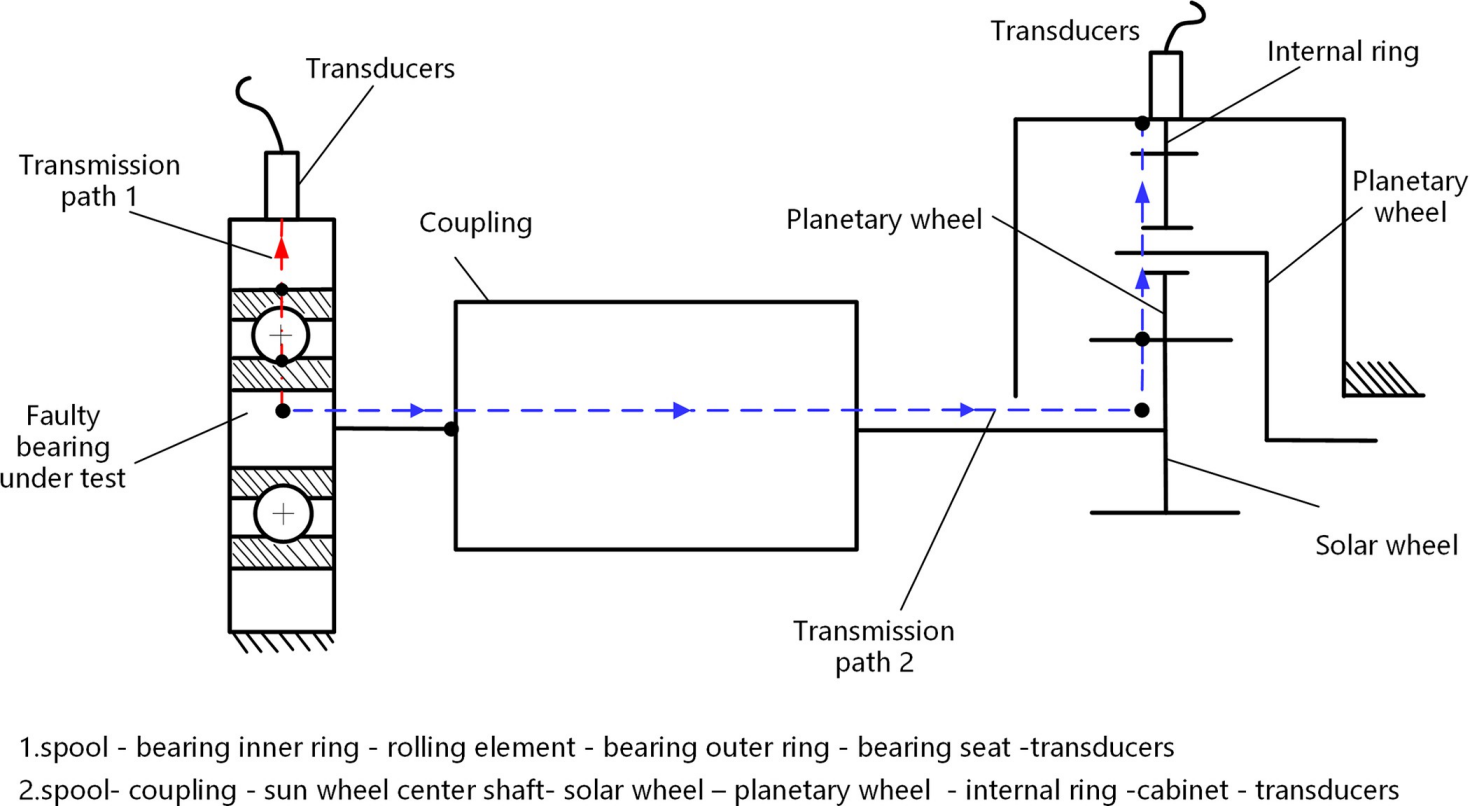
Vibration signal transmission path diagram in the planetary gearbox.

The transmission path of the vibration signal of the faulty bearing is divided into two paths as shown in the Fig 10. One path is that the fault impact vibration signal reaches the sensor through the bearing seat; Another path is for the fault vibration shock signal to be transmitted from the starting point to the coupling through the rotating shaft connected to the planetary gearbox. After being disturbed by random vibration on site, it is transmitted to the sun gear through the rotating shaft, and then to the planetary gear through the meshing point. The shock vibration during the meshing process is accompanied by the transmission to the inner gear ring, and finally to the sensor through the planetary gearbox.

### 4.2 Rolling bearing compound fault signal analysis

#### (1) Rolling bearing inner race and outer race fault vibration signal modeling

To verify the influence of multi-interference factors on the bearing inner and outer races compound fault, the simulation test bench picked up experimental data as shown in the figures below.

**Fig 11:**
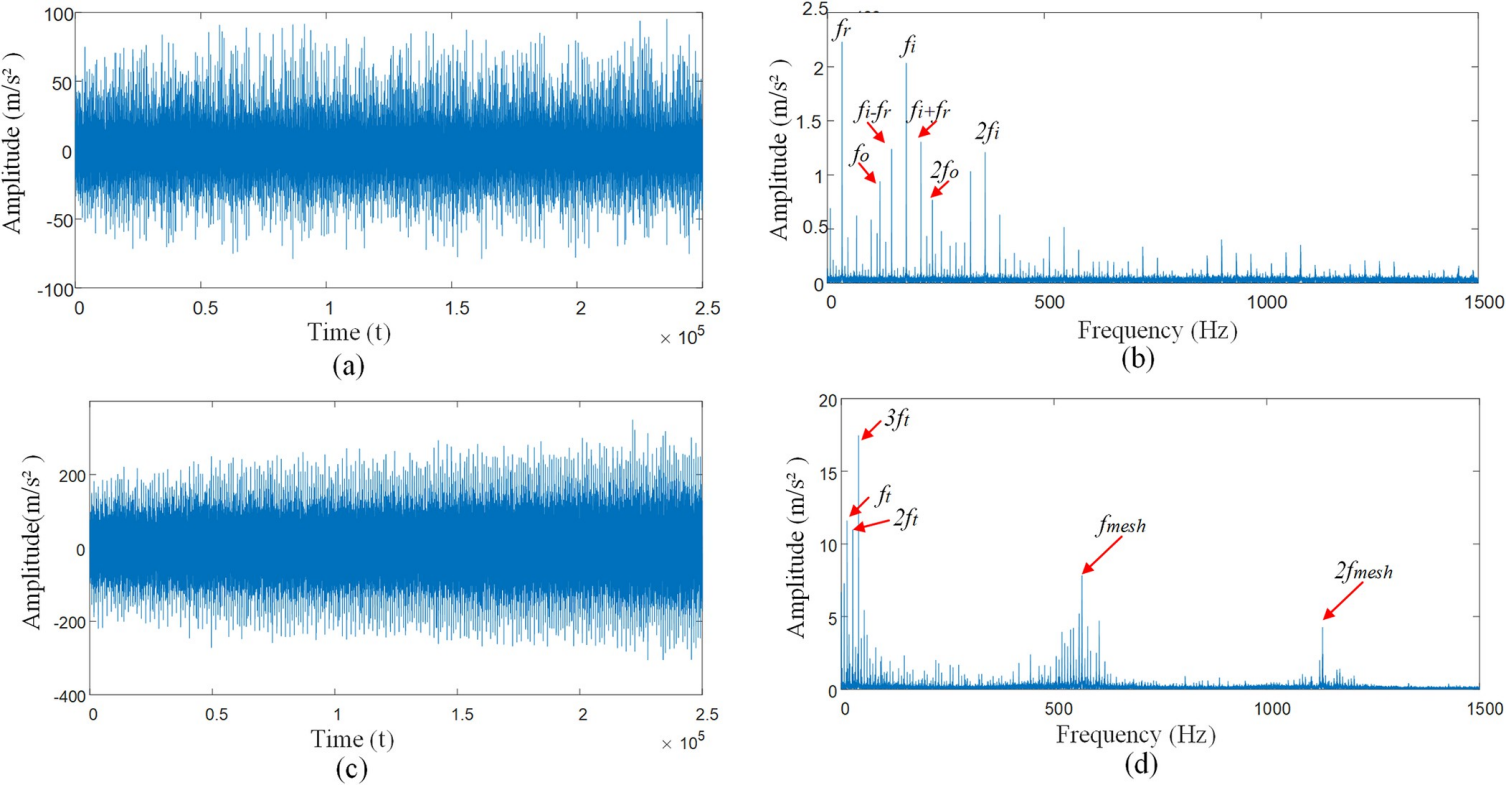
Shock response to compound fault of bearing inner and outer races. (A) Time domain diagram of compound faults of inner and outer rings. (B) Envelope spectra of inner and outer ring complex faults. (C) The inner ring is compounded with the outer ring under multiple interference factors. (D) The inner ring is compounded with the outer ring under multiple interference factors.

Fig 11 for the rolling bearing inner and outer races compound fault vibration signal impact response, from Fig 11B can be observed in the outer race of the fault frequency *f*_*o*_ and its frequency multiplier *nf*_*o*_, the inner race fault frequency *nf*_*i*_ and its frequency multiplier *nf*_*i*_, rotational frequency *f*_*r*_ and modulation frequency *nf*_*i*_±*f*_*r*_ of the inner race. As the signals of the inner and outer races are affected by the interference source, the frequency amplitude of the outer race is very low, thus showing the form of a weak fault signal of the bearing. As the inner and outer races signals are affected by the interference source, the frequency amplitude of the inner race and the outer race is very low, and the resonance bands are coupled together, therefore, in Fig 11D, it is not possible to accurately identify the fault frequency of the inner races *f*_*i*_ and outer races *f*_*o*_, and their modulation frequency *f*_*r*_. Considering the compound fault state of the inner and outer races under multiple interference factors makes the signal of the bearing as a whole show a weak fault state, and the impact characteristic frequency of the bearing is submerged under the noise interference. It can be seen that the gear meshing frequency *f*_*mesh*_ and its octave frequency *nf*_*mesh*_, the planetary carrier rotation frequency *f*_*t*_ and its octave frequency *nf*_*t*_.

**(2) Rolling bearing inner race and rolling body fault vibration signal modeling.** To verify the influence of multi-interference factors on the inner race of the bearing, the rolling body compound fault, the simulation test bench to obtain the experimental data shown in the figures below.

**Fig 12:**
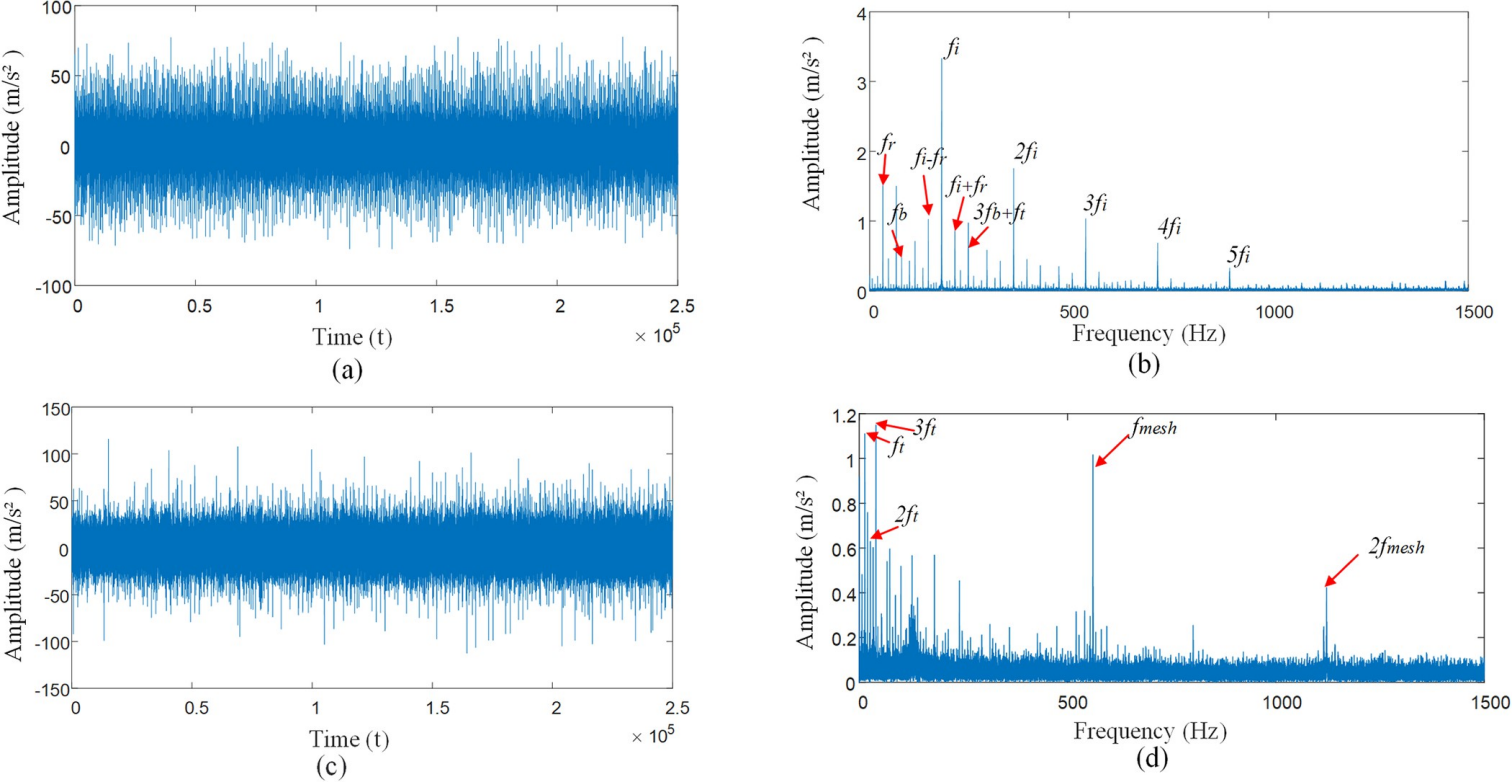
Shock response to compound fault of bearing inner races and rolling bodies. **(**A) Time domain diagram of compound failure of inner ring and rolling element. (B) Envelope spectrum diagram of inner ring and rolling element composite faults. (C) Time domain diagram of compound failure of inner ring and rolling element under multiple interference factors. (D) Complex fault envelope spectra of inner ring and rolling element under multiple interference factors.

Fig 12 for rolling bearing inner race, rolling body compound fault vibration signal impact response, Fig 12B can be observed in the inner race *f*_*i*_ fault frequency and its frequency multiplier *nf*_*i*_, rotational frequency *f*_*r*_, and the modulation frequency *nf*_*i*_±*f*_*r*_ of the inner race. Rolling body fault frequency *f*_*b*_ and its multiple frequency *nf*_*b*_, cage frequency *f*_*c*_, and rolling body modulation frequency *nf*_*b*_±*f*_*c*_. Considering the compound fault state of the inner race and rolling element with multiple interference factors, the signal of the bearing as a whole shows a weak fault state, and the impact characteristic frequency of the bearing is drowned out. The signal is affected by the interference source, resulting in its frequency amplitude being very low, so that the vibration signal amplitude relative to the noise interference is small, thus showing the bearing weak fault signal form. Through Fig 12D envelope spectrum can be seen gear meshing frequency *f*_*mesh*_ is much higher than the bearing fault frequency, so that in the low-frequency region can only be observed in the inner race fault frequency *f*_*i*_, rolling body fault frequency *f*_*b*_ rolling body and cage modulation frequency 2*f*_*b*_+*f*_*c*_, high-frequency region of the fault characteristics are submerged in the interference signal can not be accurately identified.

#### (3) Rolling bearing outer race and rolling element fault vibration signal modeling

In order to verify the impact of multiple interference factors on the composite failure of the bearing outer ring and rolling element, the experimental data collected from the simulation test bench is shown in following figures.

**Fig 13:**
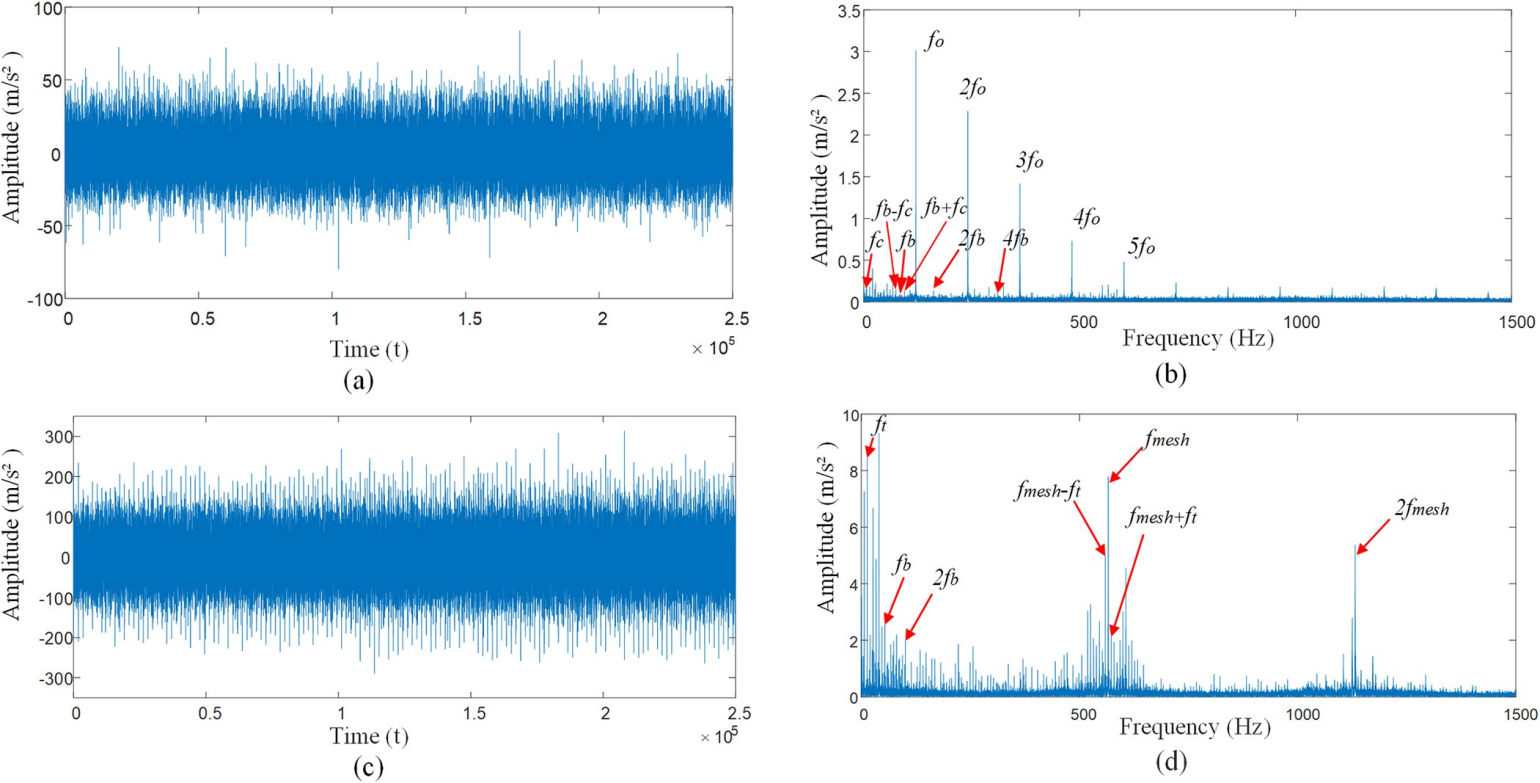
Shock response of bearing outer race and rolling element compound fault. (A)Time domain diagram of compound fault of outer ring and rolling element. (B) Composite fault spectrum diagram of outer ring and rolling element. (C) Time domain diagram of compound fault of outer ring and rolling element under multiple interference factors. (D) Envelope spectrum diagram of complex fault of outer ring and rolling element under multiple interference factors.

Fig 13 is the rolling body compound fault vibration signal impact response in the rolling bearing inner race, the Fig 13B can observe that the outer race fault frequency *f*_*o*_ and its multiplier frequency *nf*_*o*_, the rolling body fault frequency *f*_*b*_ and its multiplier frequency *nf*_*b*_, the cage frequency *f*_*c*_ and the rolling body modulation frequency *nf*_*b*_±*f*_*c*_. Considering the compound fault state of the outer race, and the rolling element under multiple interference factors, the signal of the bearing as a whole is weak, and the impact characteristic frequency of the bearing is submerged in the noise interference. Outer race and rolling element signals are affected by the interference source, resulting in very low-frequency amplitudes. The resonance bands are coupled together, so only the rolling element fault frequency *f*_*b*_ and its 2x frequency 2*f*_*b*_ can be identified in Fig 13D, the cage frequency *f*_*c*_, and the outer race fault frequency *f*_*o*_ cannot be identified, and the gear meshing frequency *f*_*mesh*_ and its octave frequency *nf*_*mesh*_can be seen.

#### (4) Rolling bearing inner and outer races and rolling element fault vibration signal modeling

**Fig 14:**
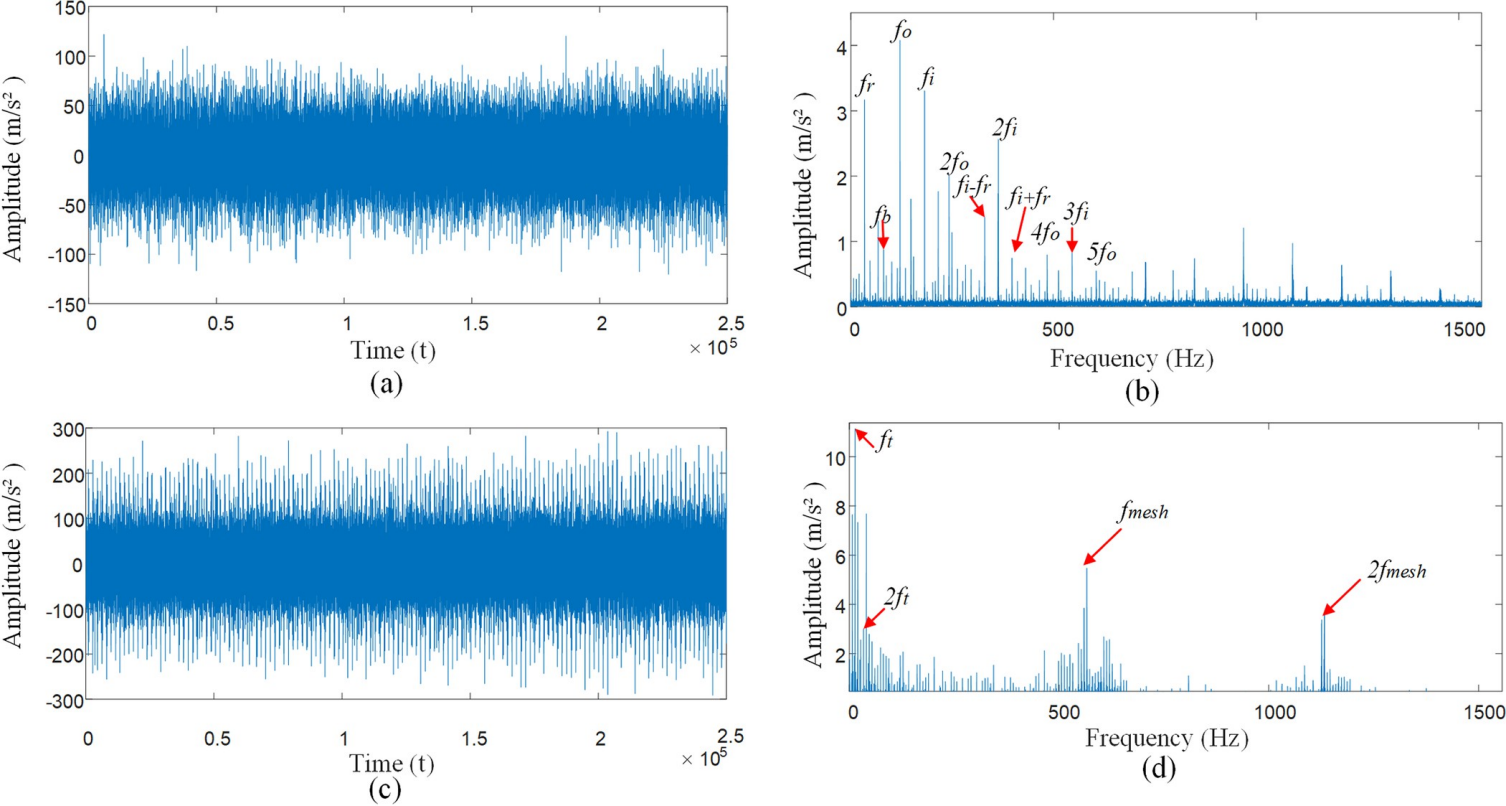
Shock response to compound fault of bearing inner and outer races and rolling bodies. (A)Time domain diagram of compound faults of inner, outer ring and rolling element. (B) Composite fault envelope spectra of inner, outer ring and rolling element. (C) Time domain diagram of complex faults of inner, outer ring and rolling element under multiple interference factors. (D) Envelope spectrum diagram of complex fault of inner and outer ring and rolling element under multiple interference factors.

Fig 14 is the rolling body compound fault vibration signal impact response in the rolling bearing inner and outer races, Fig 14B can observe the outer ring fault frequency *f*_*o*_ and its multiplier *nf*_*o*_, the inner ring fault frequency *f*_*i*_ and its multiplier *nf*_*i*_, the frequency *nf*_*i*_±*f*_*r*_ modulated *f*_*r*_ by the inner ring rotation, the rolling element fault frequency *f*_*b*_ and its multiplier *nf*_*b*_, the cage frequency *f*_*c*_, and the rolling element modulation frequency *nf*_*b*_±*f*_*c*_. The composite fault state of the inner and outer rings and the rolling element under multiple interference factors causes the overall signals of the bearing showing a weak fault state, The noise signal is mixed with the bearing fault signal, and the resonance frequency band is coupled. The frequency amplitude of the inner and outer rings and the rolling element is very low. Fig 14D can only identify the rolling body fault frequency *f*_*b*_ and its 2 times the frequency *2f*_*b*_, can not identify the fault frequency *f*_*i*_, *f*_*o*_ of the inner and outer races, and its times the frequency *nf*_*i*_, *nf*_*o*_, and the modulation frequency *f*_*r*_ of the frequency, and the gear meshing frequency *f*_*mesh*_ and its octave frequency *nf*_*mesh*_ can be seen.

### 4.3 Conclusion of the experiment

Experiments have shown that the impact response analysis of composite faults in rolling bearings under multiple disturbances can obtain that random noise interference will submerge the high-frequency part of fault information and edge frequency components, which will lead to limitations in time-domain statistical feature indicators and make it difficult to extract fault features of composite faulty bearings. Bearing failure simulation experiments through the planetary gearbox, the shaft rotation frequency and gear meshing frequency, as well as the noise masks the impact characteristics of the bearing, and the composite failure of the bearing makes the resonance bands coupled together, resulting in the inability to accurately identify the failure frequency of the bearing.

The main vibration signal is the rolling bearing fault vibration, taking the fault of the outer ring of a rolling bearing as an example, the outer ring fault will generate impact pulses with intervals of the rolling element passing through the outer ring cycle. In theory, the impact pulses generated by the outer ring fault of the bearing generally do not have a modulation phenomenon. However, due to periodic changes in operating conditions, they will be modulated by the amplitude of the rotational frequency, the bearing outer ring failure simulation signals as follows.


$$d_{i}(t)=\sum_{i=o}^{i}a_{k}(t)\cos\left[2\pi kf_{o}t+\theta_{k}\right]$$ (10)

The gear meshing frequency and its harmonics in the transmission system, and the modulation frequency is the characteristic frequency and its harmonics of gear faults. In addition, it also includes the rotational frequency components caused by installation errors of the shaft and coupling.

$$g_{i}(t)=\sum_{i=1}^{i}\left\{a_{k}(t)\cos[2\pi kf_{m}t+b_{k}(t)+\theta_{k}]+K_{R1i}\cos(2\pi if_{r1}t+\theta_{r1})+K_{R1i}\cos(2\pi if_{r1}t+\theta_{r1})\right\}$$ (11)

where: *θ*_*k*_ is the initial phase, *f*_*m*_ is the gear meshing frequency, *α*_*k*_(*t*), *b*_*b*_(*t*) are the amplitude and frequency modulation function respectively.

And ball bearings in the process of high-speed movement, due to the rolling body by the centripetal force caused by the increase in the speed of its movement on the raceway [32,33], resulting in an increase in the frequency and the amplitude of vibration signals, the centripetal force caused by the unbalanced contact between the rolling body and the raceway, the vibration signal in harmonic components enhancement. Therefore, the drive system is affected by the centripetal force *p*_*i*_(*t*) response, resulting in a system vibration signal as follows.


$$y_{p}(t)=[d_{i}(t)+g_{i}(t)]p_{i}(t)$$ (12)

Due to the installation position of the acceleration sensor on the faulty bearing seat and gearbox box, which remains fixed during the measurement process, it is believed that the position of the sensor relative to the excitation source only generates vibration signals with corresponding attenuation of amplitude, and will not cause new modulation phenomena. The bearing failure and the impact response generated by gear meshing or shaft are superimposed in the signal and do not modulate each other. Simplify the path response and assume that the impact generated by disturbances in transmission components such as bearing faults and gear meshing triggers the same resonance response. The transmission path time-domain response *h*_*i*_(*t*) [34], is convolved with the excitation signal in the time domain. Under the interference of ambient noise and other part disturbances, when a drive train has single or multiple faulty bearings with multiple defects at different locations, the final signal composition is as follows.

$$y(t)=\sum_{i}\left[y_{p}(t)+n_{i}(t)]h_{i}(t\right)$$ (13)

where: *y*_*p*_(*t*) is the vibration response of a driveline affected by centripetal influence including faulty bearings, *n*_*i*_(*t*) is the noise interference.

The signal components collected by sensors are complex, therefore, it is necessary to develop a weak signal feature extraction and a diagnosis method that can consider the influence of the centrifugal force, separate bearing faults, and explore the hidden periodic characteristics of the signal.

## 5 Conclusions

Considering influences of multi-interference factors on rolling bearings in mechanical power transmission systems under complex working conditions, a rolling bearing compound fault vibration signal impact model is established. Compound fault bearings under multiple interference factors, fault characteristic components and gear meshing components in the spectrum present full frequency band distribution characteristics, bearing fault characteristic distribution characteristics are mainly:

1. The characteristic frequency and its multiplication frequency of bearing faults are mainly distributed below the primary meshing frequency, which are affected by the low-frequency component interference caused by the rotor. The frequency modulation sidebands are rich, and are distributed near the characteristic frequency of bearing faults and the gear meshing frequency.
2. Random noise and interference components will submerge high-frequency fault information and edge frequency components, making interference the main frequency in the measured vibration signal, affecting the collection of composite fault vibration signals of rolling bearings, making it difficult to identify the overlapping fault frequencies and accurately separate the fault information of bearings.

The vibration signal impact model of rolling bearing faults under multi-interference factors is established to provide a theoretical basis for the feature extraction and diagnosis of rolling bearing composite faults based on the weak signal processing. Current composite fault diagnosis methods have limitations under multiple disturbances, and new signal preprocessing methods need to be further investigated to enhance the ability of signal separation and removal of disturbances. Consider combining domain knowledge and machine learning methods to design feature extraction and classification algorithms specifically for composite faults to improve the diagnostic accuracy and the robustness. And combine digital twin technology to assist the feature extraction and fault classification to improve the accuracy and the reliability of composite fault diagnosis. This will promote the development of composite fault diagnosis and enhance the performance of diagnostic systems.

## Supporting information

S1 Data Original diagnosis signal at 2000 rpm. (ZIP)
